# Supplementary material for: Electrochemical Biosensors Based on Convectively Assembled Colloidal Crystals
Source: Biosensors (Basel). 2022 Jun 30;12(7):480. doi: 10.3390/bios12070480 (PMC9312794; doi:10.3390/bios12070480)
Supplement: Supplementary file 1 [file biosensors-12-00480-s001.zip › biosensors-1770961-supplementary.pdf]

Supporting Information

Electrochemical Biosensors Based on Convectively Assembled Colloidal Crystals

Amane Shiohara [1,2,3], Christopher D. Easton [2], Beatriz Prieto-Simon [4,5,*] and Nicolas H. Voelcker [1,2,3,*]

[1] Drug Delivery, Deposition, and Dynamics, Monash Institute of Pharmaceutical Sciences, Monash University, Parkville, VIC 3052, Australia
[2] Commonwealth Scientific and Industrial Research Organisation (CSIRO), Clayton, VIC 3168, Australia
[3] Melbourne Centre of Nanofabrication, Victorian Node of the Australian National Fabrication Facility, Clayton, VIC 3168, Australia
[4] Department of Electronic Engineering, Universitat Rovira i Virgili, 43007 Tarragona, Spain
[5] ICREA, Pg. Lluís Companys 23, 08010 Barcelona, Spain
* Correspondence: beatriz.prieto-simon@urv.cat (B.P.-S.); nicolas.voelcker@monash.edu (N.H.V.)

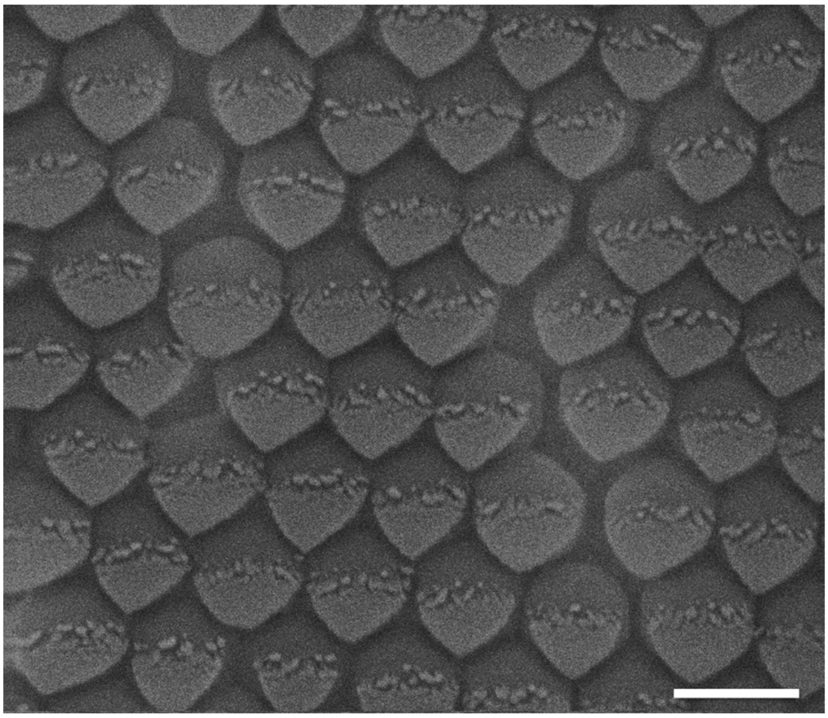

Figure S1. The SEM image shows a monolayer of the PS spheres with glancing angle deposition of silica conducted at 75° and rotation. The scale bar is 400 nm.

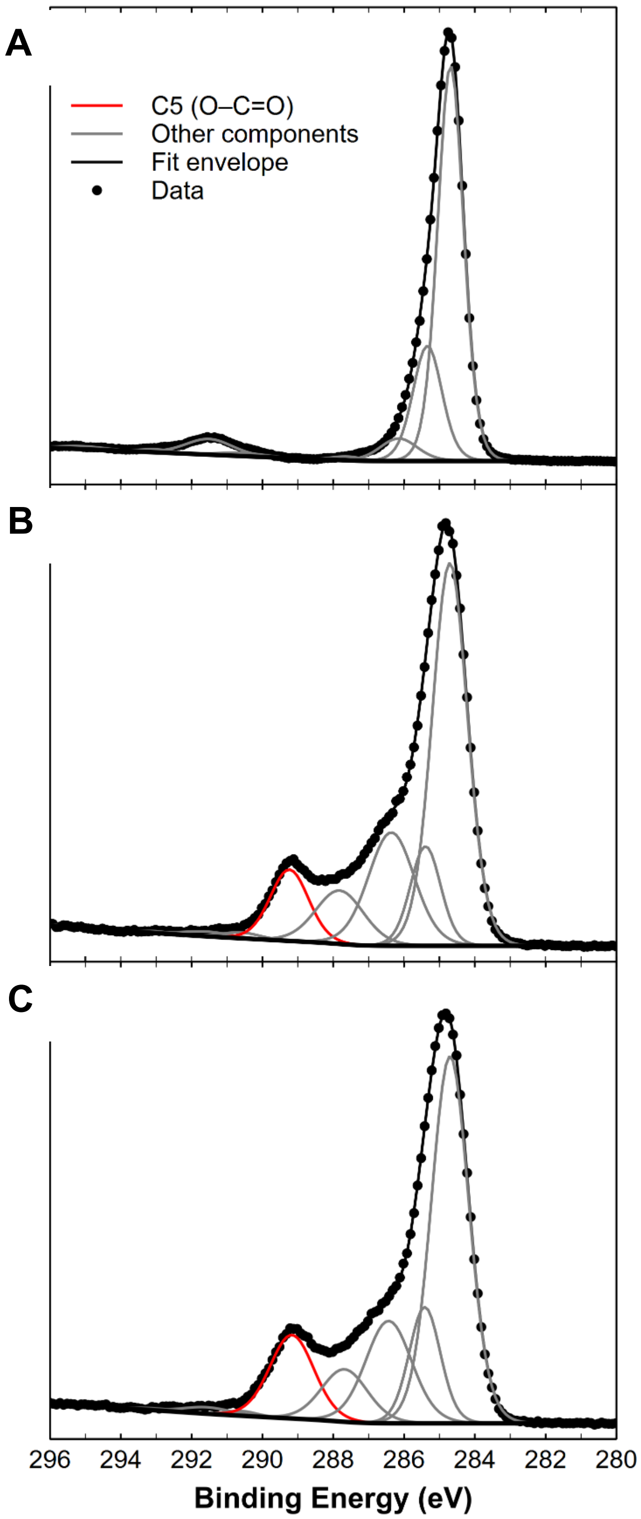

Figure S2. High-resolution C 1s spectra with peak fitting of the non-carboxylated PS sphere monolayers on gold electrodes exposed to UV ozone for A) 0 min, B) 1 min, and C) 5 min. Component C5 assigned to O-C=O groups is highlighted in red.

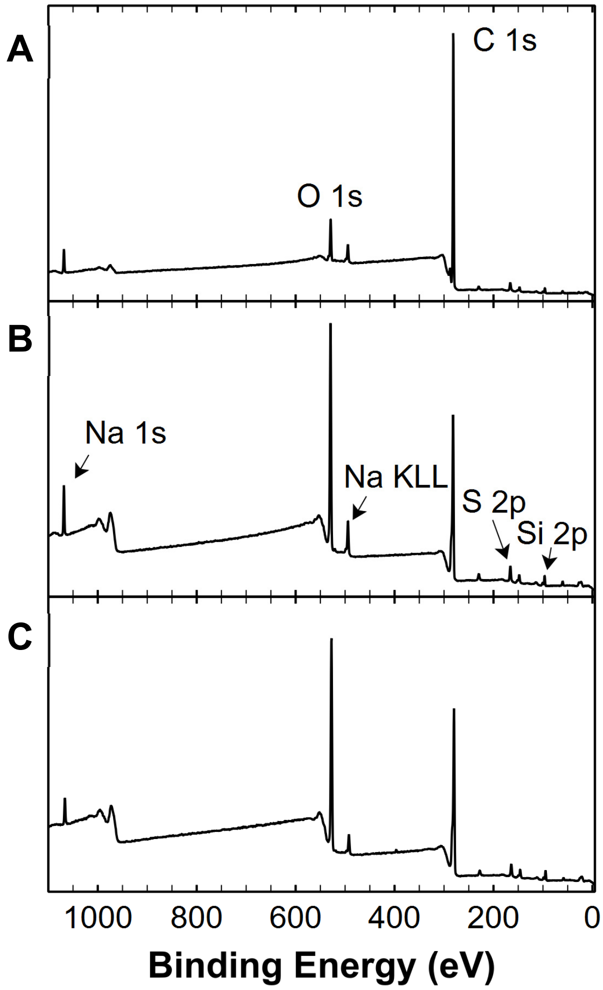

Figure S3. Survey spectra of non-carboxylated PS spheres monolayers on gold electrodes exposed to UV ozone for A) 0 min, B) 1 min, and C) 5 min.

Table S1. Elemental quantification derived from survey spectra (atomic % and atomic ratios O/C) and the amount of carbon associated with O-C=O from high-resolution C 1s spectra (atomic %, fit component C5) for the non-carboxylated PS spheres monolayers on gold electrodes from Figure S2 and S3, respectively. Results are an average of three analysis locations on a single sample.

Sample ID	C	O	O/C	C5 (O-C=O)
**No UV ozone**	89.1	6.1	0.07	0.5
**1 min**	64.1	28.2	0.44	6.3
**5 min**	66.0	27.5	0.42	6.9

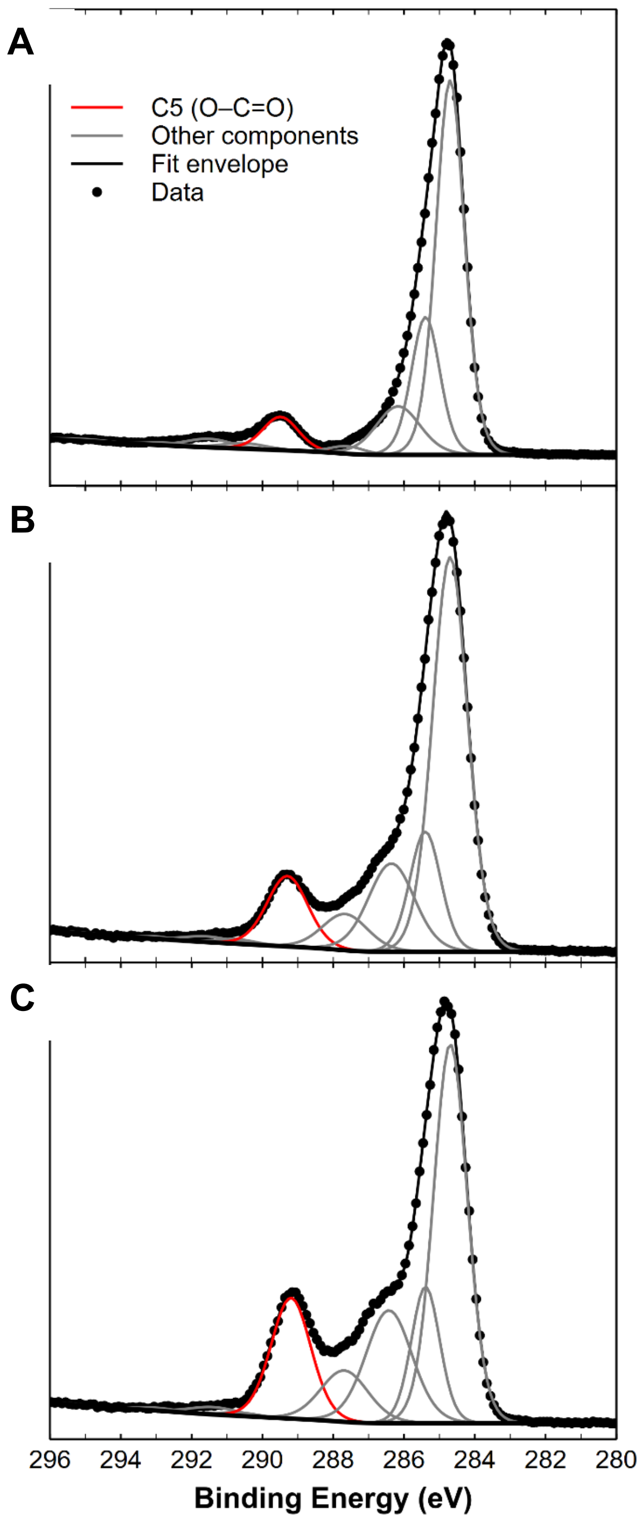

Figure S4. High-resolution C 1s spectra with peak fitting of the carboxylated PS sphere monolayers on gold electrodes exposed to UV ozone for A) 0 min, B) 1 min, and C) 5 min. Component C5 assigned to O-C=O groups is highlighted in red.

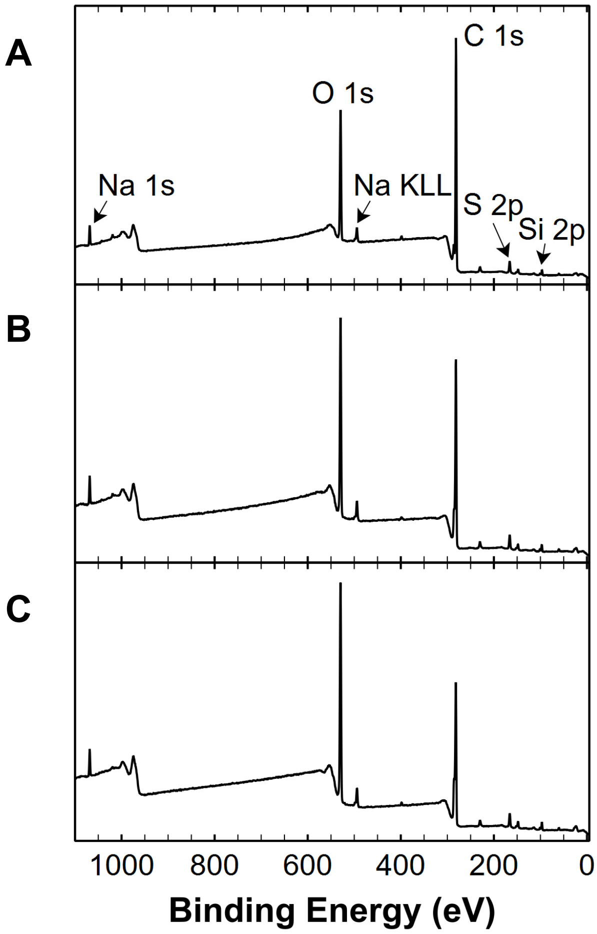

Figure S5. Survey spectra of carboxylated PS spheres monolayers on gold electrodes exposed to UV ozone for A) 0 min, B) 1 min, and C) 5 min.

Table S2. Elemental quantification derived from survey spectra (atomic % and atomic ratios O/C), and the amount of carbon associated with O-C=O from high-resolution C 1s spectra (atomic %, fit component C5) for the carboxylated PS spheres monolayers on gold electrodes from Figure S4 and S5, respectively. Results are an average of three analysis locations on a single sample.

Sample ID	C	O	O/C	C5 (O-C=O)
**No UV ozone**	76.1	18.6	0.24	4.7
**1 min**	68.1	25.8	0.38	7.4
**5 min**	62.6	30.9	0.49	10.0

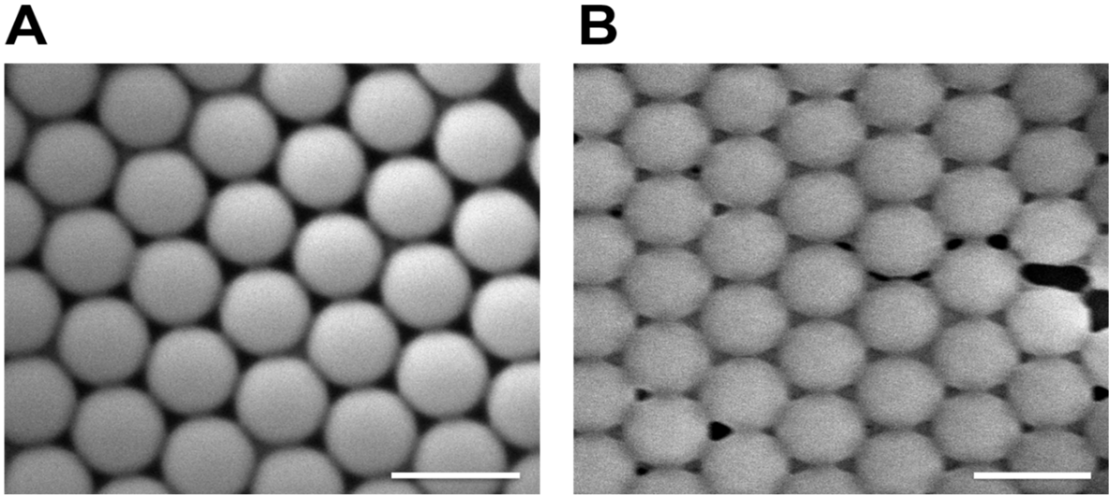

Figure S6. The SEM images of the monolayer of carboxylated PS spheres after being treated with UV-ozone for 1 min A), and 5 min B). The scale bar is 500 nm.

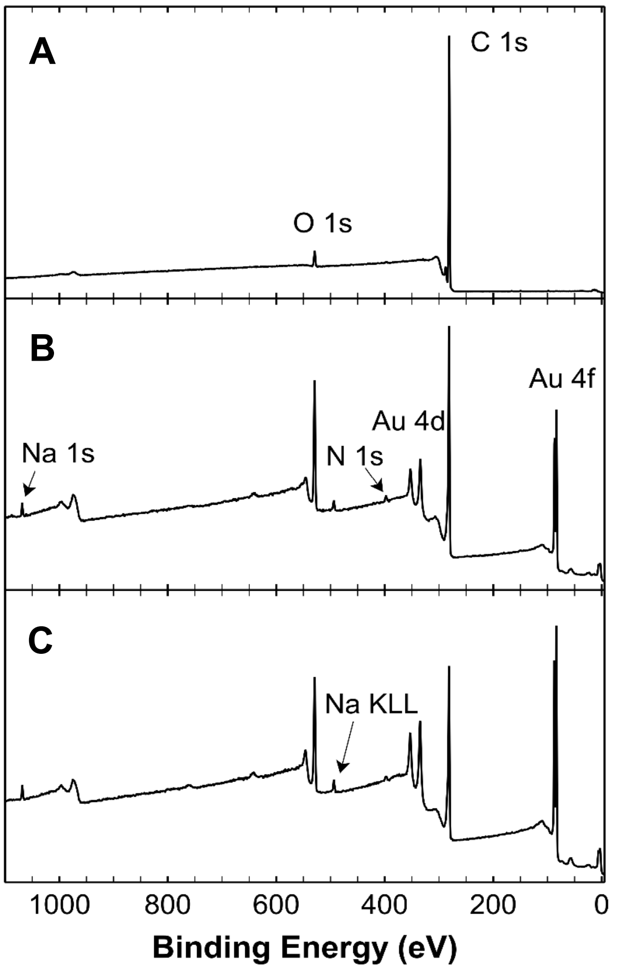

Figure S7. Survey spectra of carboxylated PS spheres monolayers on gold electrodes after A) no UV-ozone treatment, B) 1 min and C) 2 min UV-ozone treatment, all followed by EDC/NHS activation and streptavidin incubation.

Table S3. Elemental quantification derived from survey spectra (atomic and atomic ratios O/C), and the amount of carbon associated with O-C=O from high-resolution C 1s spectra (atomic %, fit component C5) for the COOH functionalised for the EDC/NHS activated PS spheres monolayers on gold electrodes from Fig S7. Results are an average of three analysis locations on a single sample.

Sample ID	C	O	N	O/C	N/C	C4 (C=O, O-C-O, N-C=O)
**No UV ozone treatment:**						
**+ EDC/NHS**	96.1	3.2	0.5	0.03	0.005	0.3
**+ streptavidin**	85.7	9.8	0.1	0.12	0.001	1.0
**+ EDC/NHS + streptavidin**	87.2	9.4	0.6	0.11	0.01	1.0
**1 min UV ozone treatment:**						
**+ EDC/NHS**	76.3	15.2	1.3	0.2	0.02	7.7
**+ streptavidin**	81.6	13.7	0.1	0.17	0.001	6.2
**+ EDC/NHS + streptavidin**	77.7	16.7	1.2	0.22	0.02	6.2

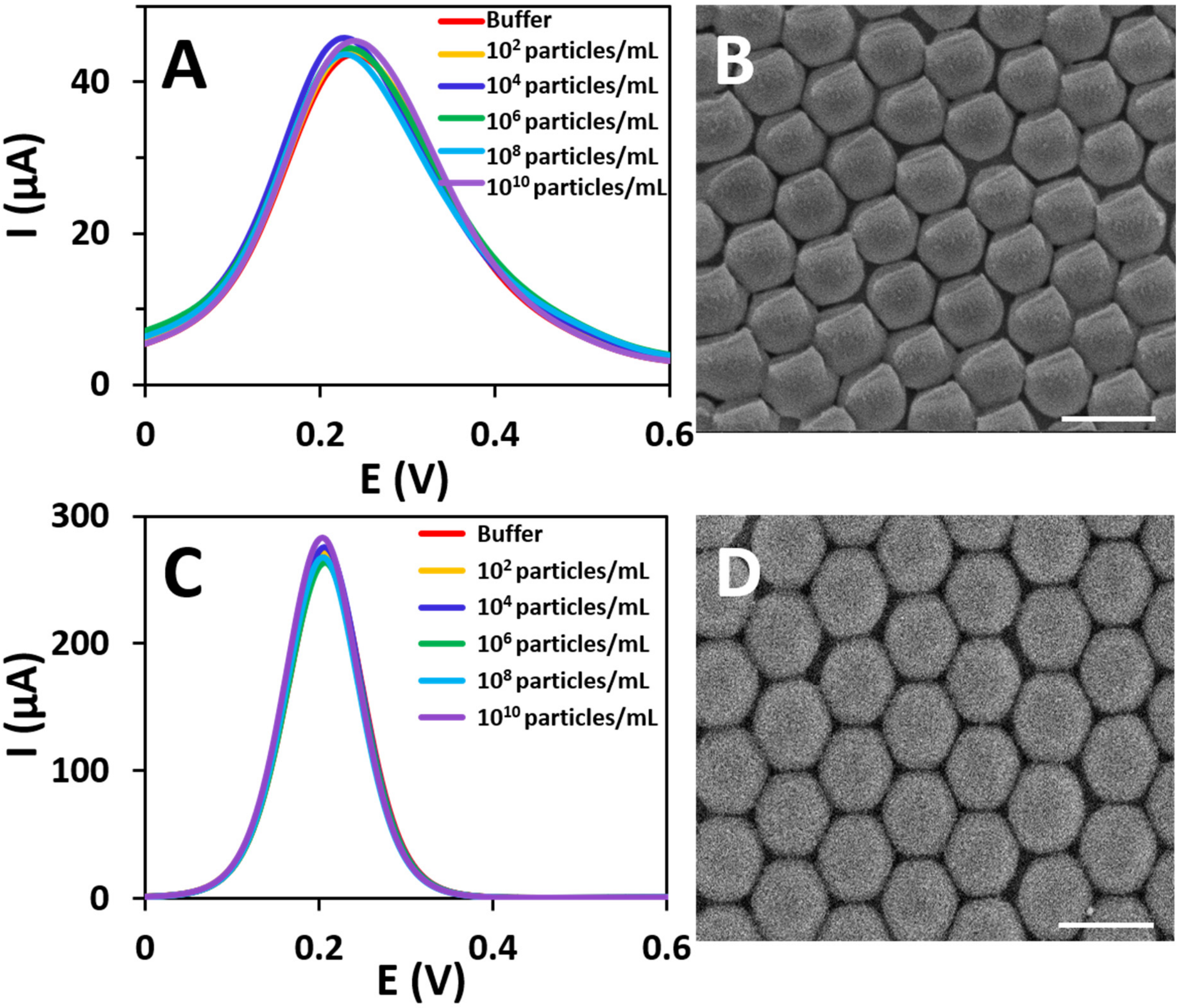

Figure S8. Differential pulse voltammograms obtained using a gold surface modified with a monolayer of streptavidin-modified polystyrene beads with A) and without C) silica deposited on top when incubating PEG-coated gold nanoparticles. The SEM images of the electrode with B) and without D) silica deposited on top after PEG-coated gold nanoparticles incubation. All scale bars are 500 nm.
